# Optimized Threshold Inference for Partitioning of Clones From High-Throughput B Cell Repertoire Sequencing Data

**DOI:** 10.3389/fimmu.2018.01687

**Published:** 2018-07-26

**Authors:** Nima Nouri, Steven H. Kleinstein

**Affiliations:** ^1^Department of Pathology, Yale School of Medicine, New Haven, CT, United States; ^2^Interdepartmental Program in Computational Biology and Bioinformatics, Yale University, New Haven, CT, United States

**Keywords:** AIRR-seq data, B-cell clonal partitioning, hierarchical clustering, optimized distance threshold, immcantation portal

## Abstract

During adaptive immune responses, activated B cells expand and undergo somatic hypermutation of their B cell receptor (BCR), forming a clone of diversified cells that can be related back to a common ancestor. Identification of B cell clones from high-throughput Adaptive Immune Receptor Repertoire sequencing (AIRR-seq) data relies on computational analysis. Recently, we proposed an automated method to partition sequences into clonal groups based on single-linkage hierarchical clustering of the BCR junction region with length-normalized Hamming distance metric. This method could identify clonal sequences with high confidence on several benchmark experimental and simulated data sets. However, determining the threshold to cut the hierarchy, a key step in the method, is computationally expensive for large-scale repertoire sequencing data sets. Moreover, the methodology was unable to provide estimates of accuracy for new data. Here, a new method is presented that addresses this computational bottleneck and also provides a study-specific estimation of performance, including sensitivity and specificity. The method uses a finite mixture model fitting procedure for learning the parameters of two univariate curves which fit the bimodal distribution of the distance vector between pairs of sequences. These distributions are used to estimate the performance of different threshold choices for partitioning sequences into clones. These performance estimates are validated using simulated and experimental data sets. With this method, clones can be identified from AIRR-seq data with sensitivity and specificity profiles that are user-defined based on the overall goals of the study.

## 1 Introduction

Next-generation sequencing technologies are increasingly being applied to carry out detailed profiling of B cell receptors (BCRs, also referred to as the immunoglobulin (Ig) receptors). Identification of B cell clones (sequences that are related through descent from a single naive B cell) from these high-throughput AIRR-seq data relies on computational analysis. Accurate identification of clonal members is important, as these clonal groups form the basis for a wide range of repertoire analysis, including diversity analysis, lineage reconstruction, and detection of antigen-specific sequences (1).

Hierarchical clustering is a widely used approach for partitioning sequences into clones (1) and several associated software tools have been developed (2–4). Identifying clonally related BCRs is typically accomplished in two steps. First, sequences are split into groups that share the same V-gene annotation, J-gene annotation, and number of nucleotides in their junction region (5–9). Here, the junction region is defined as the CDR3 plus the conserved flanking amino acid residues. Next, these groups are hierarchically clustered based on the nucleotide similarity of their junction region, and partitioned by cutting the dendrogram at a fixed distance threshold. We previously developed an automated approach for determining this threshold, and demonstrated that using this threshold with single-linkage clustering based on the length-normalized Hamming distance (i.e., the absolute count of differences between two sequences divided by the length of the sequence) detects clones with high confidence on several benchmark data sets (4). However, the actual sensitivity and specificity may differ on any particular data set, and existing methods do not provide a mechanism to estimate or tune study-specific performance. Here, we propose and validate a computationally efficient threshold inference algorithm for partitioning BCR sequences into clones that also allows for study-specific performance estimation.

## 2 Method

The proposed method extends the approach developed by Gupta et al. (4), where identifying clonally related BCRs is accomplished in two steps. First, sequences are split into groups that share the same V-gene annotation, J-gene annotation, and number of nucleotides in their junction region. Next, these groups are hierarchically clustered based on the nucleotide similarity of their junction quantified by Hamming distance, and partitioned by cutting the dendrogram at a fixed distance threshold. In this paper, we specifically develop a new model-based method for determining the fixed distance threshold for partitioning sequences, which allows for estimation of sensitivity and specificity. First, the “distance-to-nearest” distribution is determined using length-normalized nucleotide Hamming distance (i.e., the distribution of minimum distances from each sequence to every other non-identical sequence). This is typically a bimodal distribution (8, 9), with the first mode representing sequences with clonal relatives and the second mode representing those without clonal relatives (i.e., singletons) in the data set. Next, the bimodal distance-to-nearest distribution is explicitly modeled as a mixture of two univariate distribution functions (e.g., a mixture of Gaussian or Gamma distribution) of the form:

(1) $$f(x)={\text{λ}}_{1}f_{1}(x\text{|}\phi_{1})+{\text{λ}}_{2}f_{2}(x\text{|}\phi_{2}),$$

where λ_1_ and λ_2_ represent the mixing weights (summing to one), *x* represents the nearest neighbor distances, and *ϕ* represents the vector of each component parameters. Here, we investigate all combinations of *f*_1_ and *f*_2_ as Gaussian and Gamma distributions so *ϕ* is either the mean and SD (μ, *σ*) of a Gaussian distribution, or the shape and scale (*k, θ*) of a Gamma distribution. A maximum-likelihood fitting procedure (function fitdistr from MASS R package) is used to estimate the parameters of the model as follows: (1) parameters of the model are initialized using a standard Gaussian mixture model (GMM). The GMM estimates mixing weight λ_1_, mean μ*_i_*, and SD *σ_i_* where *i* ∈ {1,2} refers to the first and second distributions. (2) These parameters are then used as initial values to begin the maximum-likelihood fitting procedure (if Gamma distribution is chosen, the initial values are translated accordingly).

After fitting, the two distributions are used to estimate sensitivity (SEN) and specificity (SPC) by the fractions TP/(TP + FN) and TN/(TN + FP), respectively. The statistical rates [true positive (TP), false negative (FN), false positive (FP), and true negative (TN)] are then given by the area under the curves:

(2) $$\begin{array}{cc} \text{TP}=\int_{t_{1}}^{t}{\text{λ}}_{1}f_{1}(x\text{|}\phi_{1})dx, & \;\text{FN}=\int_{t}^{t_{2}}{\text{λ}}_{1}f_{1}(x\text{|}\phi_{1})dx,\\ \text{FP}=\int_{t_{1}}^{t}{\text{λ}}_{2}f_{2}(x\text{|}\phi_{2})dx, & \;\text{TN}=\int_{t}^{t_{2}}{\text{λ}}_{2}f_{2}(x\text{|}\phi_{2})dx, \end{array}$$

where *t*_1_ and *t*_2_ are the minimum and maximum values of the distance-to-nearest distribution, respectively. Finally, the optimized threshold *t* is chosen in the distance interval (*t*_1_, *t*_2_) to maximize the average of sensitivity and specificity:

(3) $$\underset{t_{1}<t<t_{2}}{\text{max}}\left(\frac{SEN(t)+SPC(t)}{2}\right).$$

## 3 Results

### 3.1 Mixture of Gamma Distributions Is Used to Fit the Bimodal Distribution

To determine the optimal distributions to use for the mixture model, we tested the method using simulated and experimental data sets. Specifically, we used the simulated data sets from Gupta et al. (4). These simulations start with a set of observed lineage tree topologies from lymph node samples from each of four individuals (M2, M3, M4, and M5 from Ref. (6)), and generate a simulated data set for each individual (R1, R2, R3, and R4, respectively) by randomly selecting a new germline sequence for every lineage and then stochastically re-introducing mutations along the lineage branches. This process was repeated 10 times for each individual to create a collection of 40 simulated data sets. We also invoked experimental data from BCR sequencing of PBMCs from 58 individuals with acute dengue virus infection (note that two individuals with total reads <1k sequences were excluded) (10). These samples each contained ~1–13k total reads.

**Figure 1:**
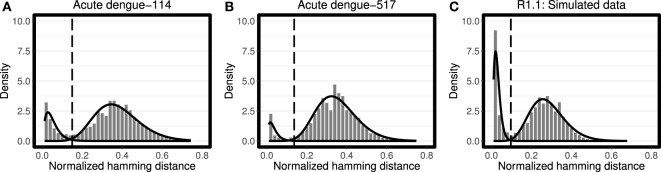
Analysis of the distance-to-nearest neighbor plot to define the distance threshold for partitioning clones. For each sequence, the length-normalized nucleotide Hamming distance to every other sequence was calculated, and the nearest (non-identical) neighbor was identified. The histogram of nearest neighbor distances is fitted using Gamma distribution for both modes (solid line) for **(A,B)** representative peripheral blood B cell samples from patients with acute dengue virus infection (10), and **(C)** representative simulated data from Gupta et al. (4). For each data set the optimum threshold, where the average of sensitivity and specificity reaches its maximum, was calculated by the findThreshold function (dashed vertical line). Note that the choice of bin size impacts the shape of plotted histograms, while the fitting procedure is independent of this bin size.

We evaluated all four combinations of Gaussian and Gamma distributions for *f*_1_ and *f*_2_ on both simulated and experimental data sets. For each combination, the log likelihood was determined once for 40 simulated and 58 experimental data sets. We found that in 80% of trials the choice of Gamma distribution for both *f*_1_ and *f*_2_ yielded the highest likelihood. Furthermore, in each trial, visual inspection suggested that this choice placed the threshold approximately equidistant between the two distributions. Therefore, Gamma distributions were selected as the default choices and used in all of the analyses below (Figures 1A–C). We note that the Gamma distribution is known to be skewed positively (i.e., an asymmetric distribution with longer right-tail). However, the Gamma distribution becomes more symmetric as its shape parameter *k* → ∞. This intrinsic feature of the Gamma distribution makes it a strong tool which behaves flexibly according to the notion of how symmetric/asymmetric the observed distributions are. By contrast, the Gaussian distribution is always symmetric, and thus unable to adapt itself to an asymmetric distribution of observed data points.

### 3.2 High Correlation Between Actual and Estimated Performance Is Achieved in Simulated Data

**Figure 2:**
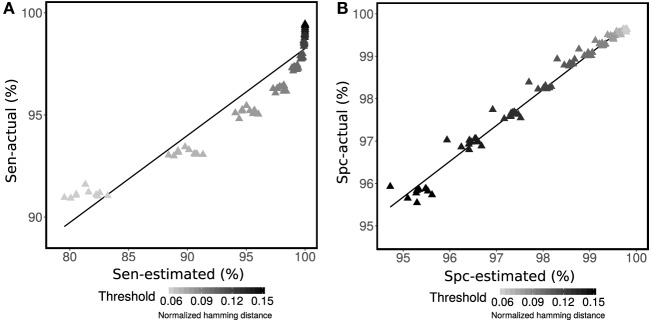
Performance assessment of **(A)** sensitivity (Sen) and **(B)** specificity (Spc) for determining membership in a multi-sequence clone. Mixture modeling of the distance-to-nearest distribution was used to estimate sensitivity and specificity for each specified value of the threshold (points) according to equation set 2. The estimated performance (Sen-estimated and Spc-estimated) was compared with actual performance (Sen-actual and Spc-actual) for simulated R1.1–R1.10 data from Gupta et al. (4) across a wide range of thresholds (shades of gray for each point).

The ability of the proposed method to estimate sensitivity and specificity for clonal relatedness was evaluated on simulated data. First, sensitivity and specificity were evaluated using ten simulated data sets (set R1 generated by Gupta et al. (4)). On each data set, a wide range of potential thresholds for partitioning sequences into clones was considered. At each threshold value, we calculated the actual performance based on the known clonal relationships from the simulation (actual), as well as the estimated performance based on the mixture modeling and equation set 2 using the area under the fitted distribution curves (estimated). We found a high correlation between the actual and estimated sensitivity (*R*^2^ = 92%) and specificity (*R*^2^ = 98%) on average over all ten simulated data sets (Figures 2A,B). We believe that the correlation is useful, as we see that method provides a lower bound on actual performance. On the other hand, sensitivity shows some lack of proportionality. Specifically, at high values for the threshold (between 0.12 and 0.15), the sensitivity estimated from the mixture model becomes saturated (i.e., the area under fitted left distribution reaches one). Although, using the positively skewed-shape Gamma distribution is better than using a Gaussian distribution, the right-tail of the first Gamma distribution still falls off too fast relative to the actual intra-clonal distance distribution in some cases.

### 3.3 High Correlation Between Actual and Estimated Specificity Is Achieved in Experimental Data

**Figure 3:**
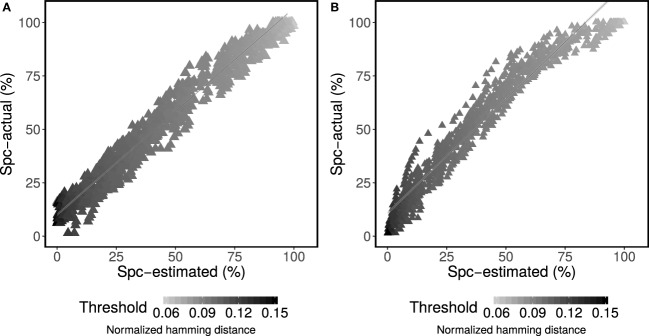
Performance assessment of specificity (Spc) for determining membership in a multi-sequence clone. Mixture modeling of the distance-to-nearest distribution was used to estimate specificity for each specified value of the threshold (points) according to equation set 2. **(A)** The estimated performance was compared with actual performance for experimental data from patients with acute dengue infection (10) across a wide range of thresholds (shades of gray for each point). **(B)** The estimated performance (Spc-estimated) was compared with actual performance (Spc-actual) across two independent experimental studies (6, 10) across a wide range of thresholds (shades of gray for each point).

The underlying clonal relationships among sequences in experimental data sets are not known with certainty. However, we reasoned that two sequences are unrelated when they are derived from two separate individuals since, by definition, a B cell clone cannot span two individuals. Therefore, false positives are defined as sequences from different individuals being grouped together in a clone, whereas true negatives are defined as sequences from different individuals that are grouped into separate clones. Specificity is then calculated by dividing the number of true negative classifications by the sum over the number of true negative and false positive classifications. We used this approach to further evaluate the ability of the method to estimate specificity on experimental BCR sequencing data from 58 individuals with acute dengue infection (10). First, one of the individuals was chosen as the “base.” Next, a single sequence was chosen randomly from each of the remaining individuals and added to the sequencing data from the base individual. Specificity was then defined by how often the sequences from non-base individuals were correctly determined to be singletons. Any grouping of these sequences into larger clones must be a false positive. Like the simulated data, specificity was calculated both using the known source of the sequences (actual) and for the mixture model (estimated). This procedure was then repeated 50 times for each of 58 different base individuals. The results indicated a high correlation between the actual and estimated specificity (*R*^2^ = 95%) across all 58 base individuals (Figure 3A).

### 3.4 High Correlation Between Actual and Estimated Specificity Is Achieved Across Experimental Data Sets

Within a single study, spurious sharing of BCRs may occur by cross clustering within the same flow cell, by contamination or by chance with low frequency. To address the possibility that these occurrences impacted our estimation of specificity, we repeated the same specificity analysis described in the previous section, but using individuals from two independent experimental data sets. First, subject M2 (with ~100k total reads from lymph node samples collected by Stern et al. (6)) was chosen as the “base.” Next, a single sequence was chosen randomly from each of the 58 individuals with acute dengue infection (10) and added to the sequencing data from the base. Like the previous analysis, specificity was then defined by how often the sequences from non-base individuals were correctly determined to be singletons, and was calculated both using the known source of the sequences (actual) and for the mixture model (estimated). This procedure was then repeated 50 times. High correlation between the actual and estimated specificity (*R*^2^ = 97%) was obtained (Figure 3B). These results show that the proposed approach provides a reliable estimate of specificity on experimental data.

### 3.5 The Mixture Method Is Computationally Efficient

**Figure 4:**
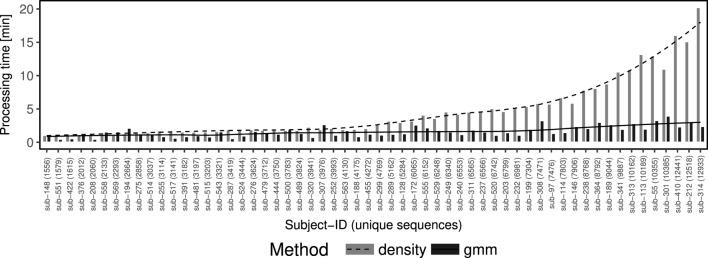
The gmm approach is computationally efficient while the density approach run time scales exponentially. Comparison of running times (*y*-axis) between density (dashed line and gray bars) and gmm (solid line and black bars) approaches performed over 58 individuals from a study of acute dengue infection (10) (*x*-axis). The *x*-axis is ordered ascending by the number of sequences in the individual sample.

The threshold inference algorithm developed in this work (gmm) is computationally more efficient than its density-based predecessor by Gupta et al. (4) (Figure 4). The improvement does not arise from the nearest neighbor identification, which is identical for both methods. Rather, the improvement comes in how to identify the fixed threshold to cut the hierarchy in order to identify discrete clonal groups. The density-based approach is computationally demanding since it is associated with a fourth derivative kernel density estimation with a sequential time complexity of O(*n*^3^), where *n* denotes the number of sequences. The gmm exhibits faster performance by replacing this computationally expensive step with an optimization algorithm with a sequential time complexity of O(*n*), where *n* denotes the number of sequences. We compared the run times of both approaches using the implementations under the findThreshold function as part of the **SHazaM** R package (version 0.1.9) in the Immcantation framework (www.immcantation.org). The density-based method by Gupta et al. (4) and the model-based method described here are implemented as methods density and gmm, respectively. On a Linux computer with a 2.20 GHz Intel processor and 32 GB RAM, we found, for example, that using the gmm approach it took <5 min to find the threshold in a data set of ~10k sequences, while the density approach completed in ~15 min (Figure 4).

## 4 Conclusion

We have proposed and validated a computationally efficient threshold inference algorithm that can be used to automatically partition BCR sequences into clonally related groups. The method gmm is based on a mixture model fit to the bimodal distance-to-nearest distribution, and allows for direct estimation of the sensitivity and specificity for membership in a multi-sequence clone. This is an important advantage over previous methods, such as the density-based method by Gupta et al. (4), which are unable to provide estimates of accuracy for new data. The ability to estimate sensitivity and specificity directly from a BCR sequencing data set allows researchers to identify B cell clones with performance characteristics that optimize study-specific goals. For instance, a threshold with high-sensitivity may be ideal for identifying sequences that are part of a clone expansion including a known antigen-specific sequence, while a threshold with high-specificity may be ideal for determining biological connections between tissue compartments or B cell subsets. In the evaluations presented in this study, we have chosen to maximize the average of sensitivity and specificity.

BCR sequencing data contain errors, although methods such as the inclusion of UMIs (11) can dramatically reduce their frequency. Thus, the distance-to-nearest distributions being fit by the mixture model contain a combination of true somatic hypermutation and errors (e.g., PCR and sequencing errors). Rather than being a problem, this is an important feature of the method. It is critical to take both sources of diversity into account when determining the threshold for partitioning sequences into clones. If members of a clone were truly <10% different, but experimental errors increased their difference to <11%, then the proper choice is to use the 11% as the threshold.

The choice of distributions (e.g., Gaussian or Gamma) that accurately describe the observed distance-to-nearest distribution for clonally related sequences in one data set may not be ideal for other sequencing data sets. The shape of the distance-to-nearest distribution depends on various experimental and physiological factors such as initial B-cell population, sampling depth, sequencing error, polarized or flat repertoire, and unusual BCR junction length distribution. These factors may influence the quality of mixture model fits. Therefore, we recommend users visually inspect the resulting fit from each data set. If a mixture of Gamma distributions results in a poor fit, then other combinations of mixture models should be tried. The density method provides a robust backup to these model-based methods, although it would be at the cost of losing the estimation of cloning performance. Our empirical observations of peripheral blood B cell repertoires suggest the bimodality of the distance-to-nearest distribution is detectable for a repertoire of minimum 1k total reads. From statistical point of view, increasing number of sequences will improve the fitting procedure, although it would be at the potential expense of higher demand in computational time complexity.

The method used in this study has been developed for partitioning BCR heavy (H) chain sequences. More specifically, the method leverages the high diversity of the H chain junction region as the main “fingerprint” to infer clonal relatedness. Emerging techniques, including single-cell sequencing, can provide paired H and L chain data (12–14). The methods presented here can be applied to such data by extending the criteria for the initial grouping of sequences to include the same V_H_ gene, J_H_ gene, CDR3_H_ length, V_L_ gene, J_L_ gene, and CDR3_L_ length. Clustering of the H chain junction region can then be carried out as before on these more refined groups. The low diversity of the L chain junction region (12) makes it unlikely that including this region in the clustering will provide a significant performance improvement.

Overall, the results on the simulated and experimental data sets indicate that the mixture modeling method provides an accurate estimate of sensitivity and specificity for hierarchical clustering-based clonal partitioning of BCRs, and is also time-efficient. This new procedure has been implemented under the findThreshold function as part of the **SHazaM** R package (version 0.1.9) in the Immcantation framework (www.immcantation.org).

## Data Access

The BioProject accession number for Parameswaran et al. (10) and Stern et al. (6) data sets are PRJNA205206 and PRJNA248475, respectively. The simulated data are accessible at http://clip.med.yale.edu/papers/Nouri2018FI.

## Code Availability Statement

Source code is freely available at the Immcantation Portal: www.immcantation.org under the CC BY-SA 4.0 license.

## Author Contributions

NN and SHK have made a substantial, direct, and intellectual contribution to the work and approved it for publication.

## Conflict of Interest Statement

The authors declare that the research was conducted in the absence of any commercial or financial relationships that could be construed as a potential conflict of interest.
